# A new electrocardiographic evaluation in hyperkalemia: frontal QRS-T angle

**DOI:** 10.1007/s11845-025-03988-3

**Published:** 2025-06-21

**Authors:** Mustafa Yedigoz, Akkan Avci, Kemal Sener, Begum S. Avci, Onder Yesiloglu, Ahmet B. Urfalioglu, Erdem Aksay, Yeliz Simsek

**Affiliations:** 1 Department of Emergency Medicine, Health Science University, Adana City Research and Training Hospital, Adana, Türkiye; 2 Department of Emergency Medicine, Health Science University, Mersin City Research and Training Hospital, Mersin, Türkiye; 3 Department of Internal Medicine, Health Science University, Adana City Research and Training Hospital, Adana, Türkiye; 4 Emergency Medicine Clinic, 25 Aralik State Hospital, Gaziantep, Türkiye

**Keywords:** Electrocardiography, Emergency department, Frontal QRS-T angle, Hyperkalemia

## Abstract

**Introduction:**

ECG is a frequently used and easily accessible diagnostic tool used to evaluate cardiac involvement in hyperkalemia patients.

**Objective:**

The present study aimed to investigate the mortality prediction potential of the frontal QRS-T angle in hyperkalemia patients and the effects of the treatment process on this angle.

**Method:**

The study was planned as a prospective study. A total of 121 hyperkalemia patients were included in the study. Conditions that caused high potassium were identified and the PR, QRS, QT, QTc, and frontal QRS-T angle were calculated and recorded from the 12-lead ECG recordings of the patients at the time of admission to the emergency room.

**Results:**

The mean age of the patients was 58.5 ± 9.2 years (median: 59), 63 (% 52.1) were female and 58 (% 47.9) were male. The second measurements of the ECG parameters PR, QTc, QRS, T-amplitude, T duration, and frontal QRS-T values were lower than the first measurements (*p* < 0.001; *p* = 0.007; *p* < 0.001; *p* < 0.001; *p* < 0.001; *p* = 0.006, respectively). The ability of the patient’s ECG parameters PR, QT, QTc, QRS, T-amplitude, frontal QRS-T angle, and frontal QRS-T (absolute value) to predict the development of mortality In the ROC Curve analysis, it was found that these parameters did not have a statistically significant effect in predicting mortality.

**Conclusion:**

As well as known ECG findings, in cases of hyperkalemia, widening of the frontal QRS-T angle and correction of this widened angle in patients with normokalemia detected with treatment can be used as an important follow-up parameter.

## Introduction

Hyperkalemia is defined as a plasma potassium concentration above 5 or 5.5 mEq/L. Normal limit values show minimal changes in the literature and different guidelines [1]. The risks associated with hyperkalemia and the severity of this condition vary depending on the rate of increase in potassium and the underlying etiological causes [2].

Acute hyperkalemia is notable for its sudden onset and often causes life-threatening symptoms because of increased plasma potassium concentration [3]. Etiological causes of hyperkalemia include impaired potassium excretion mechanism in acute and chronic renal failure (i.e., impairment of distal tubular flow, nephron loss, and renin-aldosterone system disorders), drugs that increase potassium absorption, malnutrition, insulin deficiency, and sodium–potassium ATPase pump disorders because of some drugs and potassium increase because of cell destruction [4].

In hyperkalemia conditions (potassium levels become higher than 5 mmol/L), the cardiac cells depolarize with almost no regular repolarization, which causes muscular weakness and in severe states, causes cardiac arrest in diastole [5]. Early increases in extracellular potassium levels reduce the cardiac membrane stabilization potential and lead to decreased depolarization threshold levels for Phase-0 fast Na⁺-dependent channels and increase cardiac conduction velocity [6]. These changes are detected on the electrocardiogram (ECG) as peaks in T waves, more pronounced in the precordial (V2–V4) leads [6].

With greater increases in extracellular potassium, short action potentials and prolongation of phase-4 diastolic depolarization make the stimuli delay in the AV node and His-Purkinje System more pronounced [7]. Increases in P wave amplitude, prolongation of PR interval, and widening of QRS size in advanced stages of hyperkalemia were associated with poor prognostic findings in hyperkalemia patients [8]. For this reason, hyperkalemia patients have increased risks of developing cardiac arrhythmic disorders (i.e., ventricular tachycardia and ventricular fibrillation) and cardiac conduction disorders (i.e., bradycardia, atrioventricular block, and asystole), which may be fatal [9].

ECG is a frequently used and easily accessible diagnostic tool in the evaluation of cardiac effects in hyperkalemia patients. However, ECG findings in hyperkalemia patients may be improved with treatment and cardiac stabilization can be achieved [10]. It was reported in the literature that the frontal QRS-T angle predicts mortality [11]. Although it is already known that P wave width decreases, PR interval shortens, and QRS width improves in hyperkalemia, there is no sufficient study on the frontal QRS-T angle.

In the present study, the purpose was to investigate the mortality prediction potential of the frontal QRS-T angle in hyperkalemia patients and the effect of the treatment process on this angle.

## Method

### Design of the study

The study had a prospective design. Before starting the study, approval was obtained from the Clinical Research Ethics Committee of Health Sciences University, Adana City Training and Research Hospital (Date: 01.12.2022, Meeting Number: 117, Decision Number: 2283), and patients were included in the study from December 15, 2022 to December 15, 2023. Using the G-Power program, *α* = 0.05, power (1-*β*) = 0.85, case-intervention group ratio 1:1, medium effect size, and two-sided test, the study group was calculated to include 121 hyperkalemia patients who applied to the Emergency Medicine Clinic of Health Sciences University, Adana City Training and Research Hospital.

The potassium elevation limits of the patients were found to be above 5.5 mEq/L. Conditions that caused potassium elevation were identified. Patients over the age of 18 and patients who read the informed consent form and gave their approval were included in the study. Measurements (e.g., PR, QRS, QT, QTc, and frontal QRS-T angle) were calculated and recorded from the 12-lead ECG recordings of the patients at the time of their admission to the emergency department. The frontal QRS-T angle was recorded as the value automatically calculated by the ECG device. Also, patients’ demographic data, medical history (such as additional diseases and medications), complete blood count, liver and kidney function tests, coagulation parameters, time spent in the emergency department, the clinic they were admitted to, length of stay, and hospital discontinuations were recorded.

Exclusion criteria were being under the age of 18, being diagnosed with acute coronary syndrome, having cardiopulmonary arrest at the time of presentation to the emergency department and whose spontaneous reversal was not achieved, having pre-hospital arrest and being brought to the emergency department, not having sinus rhythm on the electrocardiogram, and refusing to participate in the study after reading the informed consent form.

### Laboratory evaluation

The laboratory procedures of the study were performed in the Biochemistry Laboratory of Adana City Education and Research Hospital, University of Health Sciences. Blood samples of the participants who were included in the study and whose consents were obtained were taken from the antecubital vein in the first 10 min of their application, without using a tourniquet, as 10 mL. Control blood samples were collected half an hour after the application of anti-potassium treatments. The sex, age, and additional diseases of the patients were recorded.

The hemogram values, glucose, urea, creatinine, sodium, potassium, phosphorus, magnesium, calcium, C-reactive protein and troponin levels of the patients were measured and analyzed using the Beckman Coulter DXH800 Hematology Analyzer. Biochemical analyses were performed using the Cobas C 701 brand biochemistry autoanalyzer (Roche, Germany).

### Calculating frontal QRS-T angle on ECG

QRS and T axes are routinely reported automatically by 12‑lead standard ECG devices, and the frontal QRS-T angle can be easily calculated from them. The 12-lead electrocardiography records (GE Healthcare Mac 2000, 2063587–001, 25 mm/second, 1 mV/10 mm) of the patients at the time of their outpatient clinic application were obtained. The frontal QRS-T angle automatically calculated by the electrocardiography device was added to the data forms. Also, the frontal QRS-T angle is the absolute value of the difference between the frontal plane *QRS* axis and the *T* axis [frontal QRS-T angle = *QRS* axis − *T* axis] and can be easily calculated from a standard 12-lead surface ECG [12].

### Statistical evaluation

The Statistical Package for the Social Sciences (SPSS) 25.0 software was used for statistical analysis of the data. Categorical measurements were summarized as numbers and percentages, and continuous measurements were summarized as mean, standard deviation, median, minimum, and maximum values. The chi-square test was used to compare categorical expressions, and the Shapiro–Wilk Test was used to determine whether the parameters showed normal distribution. The Mann–Whitney *U*-test was preferred for paired group analyses in parameters that did not show normal distribution. The Spearman’s RHO correlation test was used to determine the relationships between continuous measurements.

The receiver operating characteristic (ROC) analysis was used to evaluate the effect of patients’ ECG parameters on mortality. With this analysis, threshold values were determined for each variable, and sensitivity and specificity values at these points were calculated. The statistical significance level was accepted as *p* < 0.05 in all tests.

## Results

The study included 121 patients who presented to the emergency department with a diagnosis of hyperkalemia. The mean age of the patients was 58.5 ± 9.2 years (median: 59); 63 (52.1%) were female; and 58 (47.9%) were male.

The mean vital signs of the patients were as follows: body temperature: 36.6 ± 0.4 °C; pulse: 87.1 ± 29.1 beats/min; systolic blood pressure: 125.7 ± 32.2 mm-Hg; diastolic blood pressure: 72.3 ± 19.4 mm-Hg; mean arterial blood pressure: 89.9 ± 21.8 mm-Hg; and oxygen saturation: 93.3 ± 5.9. Laboratory data of the patients are summarized in Table 1.

**Table 1:** Laboratory results of patients

	*Mean* ± *SD*	Med (Min–Max)
Glucose	173.2 ± 107.5	131 (34–671)
Creatinine	5.33 ± 4.4	4.06 (0.63–25.7)
Urea	154.9 ± 82.4	140 (12–496)
Sodium	133.4 ± 6.3	134 (107–147)
Potassium	6.97 ± 0.8	6.9 (5.58–9.9)
Calcium	8.78 ± 1.1	8.9 (5.9–12.1)
Ionized calcium	1.21 ± 0.9	1.13 (0.7–11.8)
Hs-Troponin-I	7.20 ± 4.1	6 (1–16)
Phosphorus	4.56 ± 2.3	4.1 (1.7–12.9)
Hematocrit	29.2 ± 6.8	28.8 (15.3–50)
C-reactive protein	71.2 ± 89.4	34 (1–550)
Magnesium	1.94 ± 0.4	1.9 (1.0–3.4)

*Mean* average, *SD* standard deviation, *Med* median, *Min* minimum, *Max* maximum

The second measurements of ECG parameters (i.e., PR distance, QTc distance, QRS duration, T wave amplitude, T wave duration, and frontal QRS-T values) were lower than the first measurements (*p* < 0.001; *p* = 0.007; *p* < 0.001; *p* < 0.001; *p* < 0.001; *p* = 0.006, respectively). No significant differences are detected between the first and second measurements of the other parameters in Table 2 (*p* > 0.05).

**Table 2:** The differences between the first and second measurements of ECG parameters

	First measurement	Second measurement	*p*
	*Mean* ± *SD*	*Mean* ± *SD*	
PR distance	160.1 ± 39.3	146.1 ± 33.0	< 0.001*
QT distance	385.8 ± 89.4	378.9 ± 57.9	0.007*
QTC	446.7 ± 77.6	457.9 ± 52.2	0.131
QRS duration	105.5 ± 33.2	97.7 ± 28.4	< 0.001*
T wave amplitude	3.74 ± 2.6	2.25 ± 1.2	< 0.001*
T wave duration	140.7 ± 55.4	111.7 ± 42.2	< 0.001*
Frontal *QRS-T* axis	− 40.8 ± 100.9	− 65.1 ± 91.4	0.006*
Frontal QRS-T absolute value	86.1 ± 66.1	90.09 ± 66.6	0.394
Corrected Frontal *QRS-T* axis	80.2 ± 56.8	82.6 ± 54.9	0.532

Wilcoxon sign-rank

*p < 0.01

A total of 13 patients died (10.7%). Medical treatment was given to all patients, and hemodialysis treatment was given to 72 (59.5%) patients. The average time the patients spent in the emergency department was 9.29 ± 6.5 h (median 8), and the average time to return to normal was 196.9 ± 175.5 min (median 180).

## Discussion

Hyperkalemia is one of the emergencies that threatens life and frequently causes emergency room visits. The monitoring of hyperkalemia is very important, and its vital effects occur through cardiac arrhythmias. Therefore, the ECG findings of the patients are very important. The ECG parameters monitored today are not sufficient to make an adequate and effective diagnosis. In addition, we think that the QRS-T angle can be a suitable parameter for both diagnosing patients and monitoring hyperkalemia treatment.

Increased potassium causes increased resting membrane potentials, prolongs depolarization, and causes conduction disorders, which have various reflections on the ECG. For this reason, rapid recognition of hyperkalemia and evaluation of the ECG to show the presence of cardiac effects are of great importance. It is widely known in the literature that when the K level is between 6.5 and 7.5 mEq/L, there is prolongation of the PR interval, T wave peaking, and shortening of the QT interval, while at higher concentrations, flattening of the P wave, widening of the QRS, and QRS complex disruption in the sinusoidal pattern can be detected [13].

Depending on the characteristics of the population studied, the frequency of hyperkalemia might vary. Previous studies examining patients with chronic kidney disease (CKD) reported a significantly higher incidence of hyperkalemia. The data showing the general incidence in the population show that it is between 2.6 and 7%, but this increases to 73% in patients with chronic kidney failure [2]. In the present study conducted with 121 hyperkalemia patients presenting to the emergency department, it was found that hyperkalemia developed most frequently in the chronic kidney disease group. Smyrli et al. reported that hyperkalemia was frequently detected in patients with chronic kidney disease in a study conducted in a CKD clinic [14]. Similarly, Kovesdy et al. drew attention to the elevated incidence of hyperkalemia in patients with CKD in another large-scale study conducted with 74.219 hemodialysis patients [15]. In another study conducted by Eliaçık et al. with 661 patients, the most common cause of hyperkalemia was found to be polypharmacy, with chronic kidney disease being the second most common cause [16]. Consistent with these findings, another large population-based study conducted in the USA identified chronic kidney disease as the most important risk factor for hyperkalemia, along with the use of renin–angiotensin–aldosterone system inhibitors (RAASi) [17].

The inadequacy of all these parameters has led to studies conducted on novel ECG parameters in recent years. The frontal QRS-T angle, defined as the angle between the directions of ventricular depolarization and repolarization, is one of the newly studied parameters. The frontal QRS-T angle was defined as a novel marker of ventricular repolarization heterogeneity. In another study by May et al., they stated that widened frontal QRS-T angle in diabetic individuals is a strong indicator of long-term risk of myocardial infarction and all-cause mortality [18]. In another study, a changing frontal QRS-T angle was associated with increased cardiac morbidity and all-cause mortality in postmenopausal women [19]. Supporting previous studies, in another study, a changing frontal QRS-T angle was associated with sudden cardiac death and other morbid discontinuations [20].

The spatial QRS-T angle is quite difficult for clinicians to calculate. However, the frontal QRS-T angle is calculated and documented automatically in most of the standard 12-lead classical ECG devices [21]. The frontal QRS-T angle is the projection of the spatial QRS-T angle onto the frontal plane [22]. It can be considered an easy and objective measurement when compared to the spatial frontal QRS-T angle.

QRS-T angles that are measured with spatial and frontal methods vary depending on sex and age. Women tend to have a smaller angle compared to men. Also, the calculated angle increases with age [20]. Such a variability in the reference range creates difficulties during ECG evaluation. In our study, the pre- and post-treatment values of hyperkalemia patients were compared in order not to be affected by these changes. It was found that the second measurements of the ECG parameters PR, QTc1, QRS, T amplitude, T duration, and frontal QRS-T values were significantly lower than the first measurements.

Previous studies reported the prognostic value of the frontal QRS-T angle in different populations [20, 23]. Tastan et al. investigated in-hospital mortality and the frontal QRS-T angle in patients with COVID-19, while Tekin et al. employed the frontal QRS-T angle to demonstrate early cardiac damage in patients with carbon monoxide (CO) poisoning [24, 25]. In another study conducted by Aro AL et al., the ECG results of 10.957 middle-aged Finnish individuals from the general population between 1966 and 1972 were examined, and a widened frontal QRS-T angle was associated with sudden death because of arrhythmia and sudden all-cause mortality and no associations were reported with non-arrhythmic deaths [23]. Based on this, it can be concluded that the widened frontal QRS-T angle can predict cardiac mortality because of arrhythmia. It is already known that arrhythmic cardiac complications are the basis of mortality in hyperkalemia [14]. We also used the frontal QRS-T angle so that hyperkalemia, which is known as a preventable and reversible cause of cardiac arrest, can be easily recognized by emergency physicians, especially when there is cardiac involvement. The frontal QRS-T angle was calculated and given automatically by the ECG devices in our clinic. Not to be affected by the age- and sex-dependent changes in the reference values, the changes in the ECGs taken before (hyperkalemic) and after (normokalaemic) treatment in patients diagnosed with hyperkalemia were examined, and it was determined that the frontal QRS-T value calculated in the normokalaemic state was significantly lower than the values in the hyperkalemic ECG. Considering that the sensitivity of the currently known hyperkalemic ECG findings in indicating blood K ^+^ levels is low, the use of the frontal QRS-T angle and change may provide the opportunity for early diagnosis and intervention. The present study has great importance because it is the first and only study in the literature examining the frontal QRS-T angle in hyperkalemia patients.

## Conclusion

Detection of ECG findings in hyperkalemia and follow-up with treatment are important for clinicians. As well as known ECG findings, the widening of the frontal QRS-T angle in hyperkalemia cases and correction of this widened angle in patients with normokalemia detected with treatment can be used as an important follow-up parameter. We believe that these findings can be supported by multicenter studies with longer follow-up periods and the inclusion of more patients.

## Data Availability

Data and materials are reachable from hospital automation information systems.
